# Supplementary material for: SimpliPyTEM: An open-source Python library and app to simplify transmission electron microscopy and in situ-TEM image analysis
Source: PLoS One. 2023 Oct 5;18(10):e0285691. doi: 10.1371/journal.pone.0285691 (PMC10553249; doi:10.1371/journal.pone.0285691)
Supplement: Attachment — Submitted filename: Response_to_reviewers.docx [file pone.0285691.s002.docx]

**Comments to the Author**

**Author replies are shown in red.**

**Reviewer #1:** The manuscript presents a Python library dedicated to evaluate transmission electron microscopy (TEM) images. The Python library creates metadata files, enables image processing to reduce noise, add scale bar as well as enables analysis of particles. The innovation of this work is a complete toolbox via GUI or regular coding. The current manuscript should rewritten, addressing my concerns (see attached PDF document) before its publication in PLOS ONE.

We thank the reviewer for their comments, which have improved the article. We have transcribed the comments from the attached PDF and responded to each one individually below.

Line 26: I do not think that the mentioning of the function is required in the abstract. I suggest to rewrite the abstract.

We have deleted the reference to the specific program used to perform the function. We are unclear as to other reasons for rewriting the abstract. Please could the reviewer elaborate on specific points to change in the abstract if it requires rewriting?

What is this about: EM is an emerging field

We thank the referee by pointing this out. Here we are referring to *in situ* EM, and we have added to this line to clarify and elaborate:

*Line 23: “Additionally, SimpliPyTEM specifically focuses on in situ TEM videos, an emerging field of EM involving the study of dynamic processes whilst imaging.”*

Line 59: ‘*Painstakingly*’ - Avoid the use of "literature" writing. Make simple statements

We apologise but we are uncertain as to the meaning of this comment. We have removed the word painstakingly though.

Line 62: ‘*produce a presentation to view or share these*’ - Why this is important?

Post-experimental evaluation of the images, either individually by the experimentalist or with colleagues, is an essential step of the imaging process and is commonly made easier by documents to present imaging results clearly. With this aim, we have produced a method to simply and rapidly produce documents i.e. pdf showing the images collected within an imaging session. We have added this explanation to the statement to clarify what we meant in the manuscript.

*Line 60: It can also be time-consuming to examine the acquired images as a whole or simply to produce a presentation to view and share these, for example for discussing the results with colleagues.*

Line 67: The introduction section should also be rewritten. The authors should describe the technique, including the common imaging processing tasks. Then they should discuss the current methods to provide them.

We do not clearly understand what reviewer 1 means by this comment. As we understand it, the introduction is currently structured (per paragraph) as:

1. Electron microscopy and challenges with noise
2. Requirements for image processing tasks
3. Other methods/programs to perform the image processing tasks
4. What we are introducing.

Although our proposed package has a specific focus on in situ TEM, we intend for the package to be used by standard TEM imaging as well when needed. Could the reviewer please elaborate further on what changes should be implemented here?

It is encouraged to summary the differences in a Table to show the technological need that SimpliPyTEM libraries meets.

We thank the reviewer for the suggestion and have produced a table showing some of the image-processing tasks available with the GUI and code. This can be found on page 5 of the manuscript (table 1).

Line 72: “*this macro language has a steep learning curve, is poorly documented and not transferable to other system*” - I am not sure if I agree with this statement. The authors should provide more example if the claim is the steep learning curve. I think that this may also be true for Python.

We thank the reviewer for pointing this out, we agree that the original claim was poorly phrased and not fully representative of our views. This section is also crucial to define the aims of our package, so it is important we are clear in our descriptions. We have changed this line to:

*Line 72: “However, the scope for automating complex tasks is limited and the most powerful image analysis tools are not available with ImageJ.”*

As for the reviewer’s comment of Python having a steep learning curve, this can certainly be true, however, there are many more online resources to aid in learning Python. One aim of our code library and documentation is also to lessen the challenges involved in learning to use Python for image analysis, so users can perform many powerful functions easily. We have added a line in the manuscript to point this out:

*Line 86: “Whilst Python can be difficult to learn, the wide range of users and tutorials available freely online can aid the learning process.”*

And have a line in the following paragraph to state that lowering the barrier to start using Python for image analysis is an aim of our project:

*Line 96: “The Python library aims to build on available methods by lowering the barriers of users new to Python for analysing EM images and videos.”*

Line 75: ‘*methods and modules’* : Please consider to replace methods and modules by functions and libraries/packages

We thank the reviewer for the feedback provided on these sentences. We have adjusted the text as suggested:

*Line 91: “Many image analysis libraries and functions are available with Python”*

Line 80: Try to simplify the differences between ImageJ and Python. I think that the authors should consider who are the end users. For example, in microscopy, biologists prefer ImageJ than any library than Python. Moreover, as I am going into Python, I realized that scripts may not run if the versions of the libraries are not the proper one. The authors should provide the versions for which SimpliPyTEM works.

We thank the reviewer for this comment, as it is a crucial point for defining the aims of our library, we have also started to address this in response to the comment on line 72.

We do not mean to detract from ImageJ as it is a highly effective program which we have widely used for our own analysis. However, we have experienced many flaws with using ImageJ, which are not present, or can be overcome by using Python. One example is the time taken for imageJ to open a Digital Micrograph image stack, which is often longer than the time to run our full processing pipeline in Python. We do not wish list our individual grievances in the manuscript as these are likely specific to us. Therefore, we have focussed on the key points, that it is often slower, with a less powerful scripting language and worse documentation, when compared to using Python. Python is also great because it is commonly used for some of the most innovative and powerful image analysis methos available.

As the package can be installed with Python’s package manager ‘pip’ the correct versions should automatically be installed if the installation instructions are followed in the documentation (3 commands within the command line). To ensure this, and in response to a comment from the second reviewer, we have pinned a specific version for most of the packages to ensure it will continue to function correctly. By using ‘pip’ the user does not need to worry about software versions, and the working versions can be updated with each update of the SimpliPyTEM package.

The versions of each software included with the latest version of SimpliPyTEM (1.0.6) are: python=3.8, numpy=1.24.2, ncempy =1.10.0, Pillow=9.4.0, mrcfilee=1.4.3, moviepy=1.0.3, airium=0.2.5, matplotlib=3.7.1, opencv-python=4.7.0.72, tifffile=2023.3.21, notebook=7.0.0b2, scikit-image=0.20.0, imutils=0.5.4, fpdf=1.7.2, PyQt6=6.5.0, pandas=1.5.3, seaborn=0.12.2

Line 90: Typo. Also, I do no think that the comma is needed.

We thank the reviewer for pointing out this typo, we have added a hyphen to reduced-size (*line 114)*, we believe this sentence is now grammatically correct:

*Line 93: “The app aims to allow effective image processing from large file-size images or video files in various formats to reduced-size, high-contrast JPEG files with scale bars.”*

Line 100: The current sections are a little confused since there is a significant overlap in writing between Methods and Results section.

I recommend that these two sections are combined into a section named "SimpliPyTEM: Functions and usage". The authors should briefly summarize what they are presented. It is encouraged to show a table to guide/streamline the story. It is important to highlight the libraries required as well as if there are user-defined functions that the authors have implemented.

We agree with the reviewer on this remark and have accordingly adjusted the structure of the article to address this comment. The text is presented now with a ‘Results’ section which addresses the key takeaways, including the general usage and items that have been introduced. Following the ´Results´ section a ‘Detailed methods’ section has been included, which addresses many of the individual functions and the libraries used in detail. While this may be repetitive, we believe this structure should highlight critical features of the library presented earlier with specific details provided later in the text. We hope this change addresses this concern adequately.

The authors should show examples of scripts like in the website.

While we appreciate the suggestion, we would like to avoid filling the article with too many code examples, instead focussing on highlighting the key features; the users can always find these example scripts on the documentation website.

Line 126: This sentence is repetitive - see line 114. Simplify all these paragraphs. Think that less can be more.

We apologise for the confusion here. The two sentences highlighted in these comments are intended to imply different meanings. Line 114 is highlighting the file types opened by the software, while line 126 is referring to outputted file types, i.e. those that can be saved. This is described in the text.

*Line 244: “SimpliPyTEM supports the opening of various image and video format files.”*

*Line 256: “Several choices are available for outputting image and video files from the software.”*

Line 141: Please be consistent with the verb tense. Some previous sentences were written in Present Simple and this is Past Simple

We thank the reviewer for spotting the lack of consistency in the text with regards to the verb tenses. The tenses have been adjusted accordingly. :

*Line 284: “The project herein presented aims to create…”*

Line 142: Revise verb tense

This revision has been done and changes have been implemented accordingly.

*Line 185: “To achieve this aim, we create PDF files…”*

Line 145, 149: Add as reference

These have now been added.

Line 153: Show example

We apologise for any confusion here, this section shows how images and videos are displayed within a Jupyter notebook or another Python server, therefore the examples in this case would simply be any example image or video. To clarify this we have changed the section title to ‘*Displaying images and videos’ (line 295)*.

Line 161: Add Table defining functions, inputs, outputs and the description.

We have added a table to show the functions. We would not wish to over-clutter the article, so more details on each individual function can be in the documentation. There is a lot of information on the exact use of each function and so this can be found in the lengthy reference pages of the website.

Line 162: Add more information of this function. What is the inputs and outputs. Is this function already implemented? If not, add information about the implementation. Show results.

We have added an additional sentence to extend on this function. However, we believe these questions were primarily addressed in the text below.

*Line 312: “This function therefore returns a contrast-enhanced version of the image or video, while the degree of this enhancement is controlled by the saturation value.”*

Line 172: Add more information of this function. What is the inputs and outputs. Is this function already implemented? If not, add information about the implementation. Show results.

We have added an additional sentence to highlight what the inputs and outputs are:

*Line 322: “For this function, only the number of patches is required alongside the original image, and an image with more even contrast is outputted.”*

*Line 366: “For this function, only the number of patches is required alongside the original image, and an image with more even contrast is outputted.”*

We are hesitant to include excess unnecessary figures and so have shown an example here. However, a detailed example is included, via the link to the documentation, in the text.

Line 181: Add this as a reference. Note that this website has so much information that the manuscript. I think that this information should be the same.

We disagree with the reviewer on his/her remark stating that ´this information should be the same¨, as the website documentation has no limit of size, while a journal article benefits from being shorter than a website. We also believe that an article and a website serve different purposes, as a journal article advertises what can be done with the package and how this has been implemented, while the documentation serves to tell the user how specific functions can be used directly in a more hands-on practical fashion. The documentation is also a live document so can be updated and changed with changes in the software, whereas the manuscript is a static document.

Line 184: Add more information of this function. What the inputs are? How the function knows the pixel size? The magnification is also required? Please add more information and show results. / Line 185: Please consider the following rewriting:"The scale bar is individually chosen based on the size of the image" The authors should revise the writing, simplifying it.

As requested by the reviewer, additional information has been added for this function:

*Line 329: “A scale-bar can be easily added to an image with a single command. The size of the scale-bar is chosen as a fraction of the size of the image, meaning scale-bar sizes appear consistent for images of different dimensions. The pixel-size is taken from the metadata of DM files automatically but can also be loaded in when opening other files, or defined using a separate function (set_scale).”*

Line 197: Please add more information about the thresholding method. In the website, it is showing that the user must select the thresholding manually. Is this true? What about using the Otsu's method? Please show results and discuss.

We appreciate the simplicity of the approach and aim to implement more complex approaches in the near future, for example, implementing Meta’s Segment Anything model. However, in our experience, automated thresholding methods, including Otsu’s, have not worked well with our noisy EM data. Here we have tried to introduce a simplified method to introduce particle analysis with Python to beginner users as well as introducing methods to extract the data. The original segmentation of the images will always be the most challenging step and more complex methods are often required for this.

Line 210: Improve quality of all images. They are blurred. Numbers and font are hard to be read, increase the font size.

The images added to the manuscript here are presented in low resolution automatically by the submission process. We ask the reviewer to check the full-resolution images that were included in the submission.

Please be clear about the functions used to calculate the morphology of the particles. Consider to have a script.

We have chosen to leave the script out for the sake of simplicity. However, the complete method from start to finish is available as an online tutorial, which is highlighted in the text. A description of the steps used for this process is included in the ‘Particle analysis’ section of the manuscript, we have edited this for clarity.

*Line 250: “The process for measuring particle properties is simple and involves applying a threshold to separate the nanoparticles from the background intensity, locating the boundaries of the particles and filtering the selected particles by area. The process will yield the user to measure the physical characteristics of the particles, which are returned in a Python dictionary of characteristics.”*

Line 212: Add as a reference

We appreciate this comment, however we have opted not to add links to our own documentation as references, as this article is designed to be the citation for the library. The documentation will be linked in the article in several places to ensure it is easily found.

Line 214: ‘*Metadata*’ - Please introduce the importance of this. Maybe this should be introduce before the image processing.

This was touched upon in the introduction (*line 78*); however, as we have also now restructured the article, the value of this section is now discussed in the results section before going into detail about the functions in the detailed methods.

Line 222: This section is only important to discuss how the usage of the library has been verified. Consider adding in the beginning of the new section. Please consider to make the data open access for usage trying to use the library.

We thank the reviewer for the comment about adding the data to the repository. We have added the example images to the github repository. Full tutorials of how to process this data are included on the documentation site.

Line 228: The discussion of the GUI should be after the discussion of each previous sections. Introduce it for users without coding knowledge. Consider to provide a simple flowchart for the steps.

As requested, we have changed the order of the results section to introduce the image and video processing libraries before introducing the GUI.

Line 245: This whole paragraph is not adding any information

This paragraph has been removed as requested.

Line 252: ‘*Topaz’* Add more information about this method. Who has implemented it? It should be introduced before and clearly mentioned that it required CUDA.

We have added additional information on this, including mentioning the speed increase a CUDA GPU allows.

*Line 136: “...using the deep-learning based denoising method Topaz, produced by Bepler et al (6).”*

*Line 141: “Unfortunately, this does have significant hardware requirements and benefits greatly from a CUDA GPU for fast processing. On a Macbook Pro, 2018 Topaz takes several minutes per 32-bit 3838x3710 pixel image, while a CUDA GPU can accelerate this to mere seconds.”*

Line 259: The authors should clarify why this is important. The authors should show an example of how to doing it and the output.

We have added a line to address this:

*Line 195: “This development is beneficial as it allows for rapid sharing and discussion of the results with colleagues or collaborators. Examples of these documents can be seen in* ***Fig. 2****.”*

Example images of the documents can be seen in Fig 2 (formally Fig. 1). A video showing how this can be done will soon be available on the documentation site.

Line 266: Another sentence that has already been said

This sentence has been removed as requested.

Line 269: How is this information extracted? The authors should add more information and show an example of usage.

This information is taken from the metadata tags in the image files' header and saved into a .csv file. We have added information into the metadata section (*Line 437) on this:*

*Line 361: “Image metadata can be useful but difficult to access with TEM files. As such, we have tried to make it more accessible by saving the image or video metadata from DM files into a comma separated value (CSV) file, showing many of the key values saved within the file. The metadata is taken from the headers of DM files using openNCEM (19), from which several key values are extracted.”*

Line 286: It is recommended to use a table instead of a figure. Also, the codes should be use for each example.

We are grateful for this suggestion, we have added a table (Table 1, page 5) to show many of the functions available from the library from both the GUI and the code. Again, we wish to avoid filling the manuscript with too many code examples, as these will always be available from the documentation.

Line 294: Please be consistent. At the beginning it is said linear filters, latter median and Gaussian, then it is introduced Topaz and here there are three more. For each filter, mention if it is the function implemented within the library or a user-defined function.

We thank the reviewer for this comment about lack of consistency. We have edited the detailed methods to mention all the filters in a separate paragraph (Line 361)

Line 294: ‘*Weiner*’ - Typo

The typo has been fixed.

Line 296: This is has already been mentioned, therefore it is not added any information.

This sentence has been removed as requested.

Line 369: Add the data within the GitHub

We have now added the image data from the tutorials to the github page (docs/Tutorials/)

Line 375: Consider to record and upload YouTube videos of how to use the library, including its installation.

We thank the reviewer for the suggestion, we have recorded basic videos for installation and usage of the GUI which we plan to upload very soon. We also plan to record additional tutorial videos in the future.

Line 375: Please in the revised manuscript add sample codes for processing of TEM images as well as troubleshooting documentation. Please add appropriate error massages to guider users upon error.

We believe the manuscript and documentation should remain separate, as the documentation is much longer than a standard manuscript. The manuscript aims to explain the use and value of the software, while the documentation provides all the information needed for users to use it themselves. This separation is standard among published software. However, if the reviewer (and editor) deem it valuable and useful, could we include the documentation as an automatically generated PDF as a supplementary document?

As for appropriate error messages, we have tried our best to do this throughout the code and will continue to add further error messages for any common errors seen. We also would welcome any user who comes across an error they cannot solve to post it upon the issues page in Github and we will do our best to resolve the error.

Can external users contribute to the library?

Yes, we welcome external contributors; they will just need to create a local branch from GitHub, edit the code, and then re-upload as a pull request to the GitHub.

**Reviewer #2:**

This manuscript describes a simple package written in Python that is designed to make access to image stacks in various formats associated with current electron microscopic images. The purpose of the program is relatively simple - it is not for complex analysis, although it does some analysis, but for manipulating the images for sharing, perhaps preliminary analysis, and examination. I can see where this would be a useful tool for many labs.

We are pleased the review sees the value of this work.

One concern is that this paper does not describe scientific results, which is the first item in the list of elements for review, so it is not clear that it is a suitable fit for this journal. This is for the editors to determine. I think the work would be a stronger scientific contribution if the authors could show how it leads to or enables some scientific discovery.

We thank the reviewer for raising this issue and agree that this is for the editor to determine. Here we introduce a tool to improve the data-processing pipeline, which although is not new scientific results, does allow opportunities for faster data processing, thus speeding up scientific discovery.

We would like to note that on the PLOS ONE submission guidelines, submissions presenting new software are included as: *“PLOS ONE will consider submissions that present new methods, software, databases, or tools as the primary focus of the manuscript if they meet the following criteria…”*

As for demonstrating scientific discovery, the software has proved very useful in a number of our projects, including one submitted manuscript, however we wanted to keep the different focuses of the manuscript separate.

The paper is clearly written, although in places it is somewhat repetitive - in particular the results text recapitulates some of the methods section.

We have edited the manuscript, including restructuring the article, to improve the flow and decrease the repetition, we hope this has improved these issues.

The documentation (on [readthedocs.io](http://readthedocs.io/)) is fairly good, although there seems to be some disorganization (opening the "micrograph class" has a link to the "micro video class", which is actually the next element. the list of functions in the "Documentation" is a repeat of the "List of functions..." in the link above it. This should be cleaned up. The tutorials look nice however.

We are grateful the reviewer took the time to browse the documentation and appreciated it. We believe we have corrected the mistakes which the reviewer highlights, and we welcome any additional comments highlighting other errors. We take great pride in making the documentation as accessible as possible to make it easy for new users to learn.

In general I am not a fan of using JPEG for image rendition, as it can introduce distortions, notably in the color space. It is probably ok for this particular use case, but the generation of output files should be flexible and include formats that do not involve the kinds of compression that can distort subtle aspects of an image. I think this should be empahsized more in the methods section.

We appreciate and agree with this concern. For general usage, we find that JPEGs are generally adequate for display purposes, which is the primary aim for the GUI, whilst for further analysis, uncompressed formats like Tifs are ideal.

Whilst the ability to save in multiple file formats has always been present by coded means, we have introduced the choice of output filetype (32-bit tif, 8-bit tif and JPEG) within the GUI so users can choose an uncompressed filetype if they wish, including keeping the file as close as possible to the original (as a 32-bit tif).

We have also added a line in the methods to address this matter:

*Line 260: Saving images as JPEGs can introduce subtle distortions in the image, but can be useful for ensuring files remain lightweight for easy viewing and sharing.*

line 82: Many languages are faster than Python (C and it's derivatives, and Julia, for example). Whether the code is accessed by scripting (command line) or from a gui is not really relevant here; the great advantage of a gui is user convenience and generality, and it should have little effect on the actual processing speed.

We have rephrased this paragraph to address this remark. In our opinion, Python hits a sweet spot regarding usability, popularity and processing speed.

*Line 75: “Coded approaches can be very effective,* ***and the most popular language since October 2022, is Python (10), Python is also widely used for science and image analysis.*** *Many image analysis libraries and functions are available with Python, including openCV (11), pillow (12) and scikit-image (13), making it possible to perform an enormous variety of tasks. One advantage of using Python for this analysis is the prevalence of Python-based machine learning and deep learning-based image analysis tools (14, 15), which commonly use images in a similar format* ***and can easily be combined with other Python-based workflows.*** *At the same time, the range of available options and locations can make it difficult for newcomers to locate the required methods. Many of the functions in these libraries also come with many parameters which can make the function much more complicated to use than is necessary for most cases. The upside of using Python is huge however, Python is much faster for many tasks than ImageJ, and scripting can automate image analysis processes to reduce the amount of manual effort.* ***Whilst Python can be difficult to learn, the wide range of users and tutorials available freely online can aid the learning process.*** *While scripting tends to be more powerful than user-interface based approaches* ***due the wider availability of options****, many potential users find coding intimidating and challenging to learn, leading to users performing time-consuming manual methods.*

Code:

1. In several locations, "from xyz import *" is used. This is generally bad practice; use explict imports ("from xyz import abc", or "from xyz import abc as ABC"). Explicit is always better than implicit (Zen of Python).

We have changed this in several places and will continue to correct any further examples we find, we thank the reviewer for introducing us to this concept, as it will improve the readability of our code.

2. The code presentation would benefit from some reformatting (using "black" and "isort").

Again, we are very grateful to the reviewer for introducing these methods to us and have used these to improve our code.

3. The program uses PyQt5 as the gui backend. This has been superseeded by PyQt6. PyQt5 (as provided from the vendor) does not always work on Apple M1/M2 processors, and I was unable to get the code to even install using the instructions on readthedocs (macos, M1 processor). The documentation warns about this. It should not be hard to update this to PyQt6 to enable this functionality, given that most of the gui code appears to be in one fairly short file. This could extend the lifetime of the code, as well as its reach to other labs.

We thank the reviewer for this helpful suggestion. This was easy to implement and has been uploaded in the most recent version of the software (1.0.6). We hope that with the implementation of PyQt6 there will be less installation issues. If there are any further issues we would be very willing to trouble shoot.

4. The requirements.yaml (and setup.py) files did not specify versions for most of the imported libraries. It can be useful to "pin" these to specific versions to ensure that the program will run in the future when the libraries might be updated with incompatible requirements or fixes to their code. For example, the documentation mentions Python 3.10, but the condo install instructions point to Python 3.8.

We thank the reviewer for the suggestion. We have implemented pinned the versions of most of the libraries in setup.py to ensure the library continues to work the required libraries are updated.
