# Supplementary material for: SimpliPyTEM: An open-source Python library and app to simplify transmission electron microscopy and in situ-TEM image analysis
Source: PLoS One. 2023 Oct 5;18(10):e0285691. doi: 10.1371/journal.pone.0285691 (PMC10553249; doi:10.1371/journal.pone.0285691)
Supplement: Attachment — Submitted filename: Reviewer_reponses2.docx [file pone.0285691.s003.docx]

Reviewer #2:

The authors had done a reasonable job of revising this manuscript based on the reviews. However, there remain some significant issues. Before I can evaluate the overall ms however, the program needs to be able to run without being edited.

I remain unable to get the GUI program (as posted on GitHub) to run, which means that there is something about the structure or instructions that is not correct. Following the instructions on readthedocs, under "SimpliPyTEM-GUI", an attempt to run the program throws the error "ModuleNotFoundError: No module named 'SimpliPyTEM'". I suggest the authors try an install on a clean machine (no pre-existing conda library or pip installs), and be sure that the instructions result in a runable GUI. I also suggest including the install instructions clearly in the README.

There are 2 ways in which users will obtain this program: either directly from the github repo, or from pypi. The instructions should be clearly separated to be able to make working environments. I note that this applied to using the GUI; I suspect that using the library programically is fine however.

I finally did get the program to come up by removing the "SimpliPyTEM." from all the imports in all of the source files when running from the terminal, and changing the code a little.

Author response:

We apologise for the inconvenience the reviewer has experienced due to difficulties in software installation. The installation instructions were included on the first page of the documentation, but we have now added this to the GitHub README to improve accessibility.

We require installation using Pip to ensure that all required libraries are installed properly, including this one itself, avoiding any ModuleNotFoundErrors. Pip is one of the most common Python package managers and its use is recommended by the Python Software Foundation (<https://packaging.python.org/en/latest/guides/tool-recommendations/>). We have however included instructions to download the software from GitHub while using pip to ensure it is properly installed whilst still allowing developers to edit the code easily.

We want to emphasize however that the software has been installed on several computers by a number of different groups, and that it has regularly been successful using Pip. We have tried to reproduce this error but have been totally unable when using pip and thus without more information we cannot help here. We may be mistaken but this ModuleNotFoundError sounds as though the reviewer has downloaded the repository directly from GitHub rather than using Pip, as the installation instructions require. If the module had been correctly installed, the import statements would function correctly without any need for editing.

Upon further examination of the documentation, we do recognise how these installation instructions could be missed if users go straight for the GUI instructions, as the installation instructions were below the GUI introduction section. To rectify this, we have adjusted the structure of the documentation, so the installation instructions are closer to the top, enhancing visibility.

After that, I attempted to load a simple tiff file with the GUI. The call to "process_file" complained that there were 10 position arguments, but 12 were given in the call (this is all within SimpliPyTEM_GUI.py). The call specifies self.topaz_on and self.cuda_on, but these are not in the argument list for the function itself.

We apologise for this oversight; the error has now been remedied. This is a lesser-used function as the library is mainly used for batch processing rather than an individual file, so this error had been overlooked since a recent update. We are grateful that the reviewer brought this bug to our attention, and we encourage any user to report bugs on the GitHub issues page.

I suggest several things:

1. Use mypy or another linter to seriously lint the program. The GUI part at least is not ready for release.

We thank the reviewer for his suggestion. We have downloaded and used mypy on all the files included within the library and found a single error in the html_writer.py file which we have now corrected.

We feel the second comment is unfair, as the GUI has been used by several groups on different PCs and has been found to be effective. While the reviewer has found one minor bug which was easily fixed and has issues with dark mode which have been addressed below, the core of the program remains effective and useful.

2. Use typed arguments (hints). You may have to move up from python3.8 to 3.10 or 3.11 to fully take advantage of this. However, it would avoid the error above where the function call and the arguments did not agree. It also will be sure that the right argument is passed to the right variable in the function call, and can define values for variables that are not passed (solving the problem above).

The use of typed or untyped programming is a stylistic choice of how to develop the software, we prefer the less restrictive untyped approach as it leads to quicker development and flexibility in future development of the code. While we agree there are good reasons to use a typed approach, we have not done so here, which is a common practice in Python coding and we want others to adopt and modify this code for their own uses and labs with as few barriers to doing so as possible. We appreciate the reviewer may disagree with the choice, but don’t think stylistic choices of coding style is part of the scientific review of the work presented here.

3. The color scheme did not work well on my computer. The text in the GUI was white, the background was light cyanish, and the text in the text boxes was also white and unreadable. My computer runs in "dark mode" so text is typically white or light, but attempts to change the style within the GUI (e.g., settting to "fusion", which works with other code) did not work to make the text stand out. I am not sure the add_styles function was accomplishing its goals either.

We apologise for this error, we had not considered ‘dark mode’ changing the text, as for development operating systems (MacOS, Ubuntu Linux) dark mode has no effect on the program. Unfortunately, as such we have been unable to reproduce the error.

We have attempted to fix this by ensuring that all the text styles are set to black within the add_styles function, which we have confirmed works by changing text to other colours. We hope by defining the text colours here, we will override the system changing the text colour and therefore solve this error. We will happily re-address this issue if it has not been fixed however, if so we request that it be reported through the GitHub issues page, so we can discuss solutions more easily.

Some specific minor comments:

Line 86: , -> .

line 86: Python is not always faster than ImageJ. ImageJ can be scripted (even with Python) to automate tasks - not easy, but not hard either. I would modify this statement.

We thank the reviewer for highlighting this, we have removed this sentence.

line 93: "app" is jargon. Use either "application" or "program" to refer to the GUI, or "library" for the library functions.

We thank the reviewer for pointing this out, we have changed this.

lien 294: "PyFPDF" is no longer maintained according to their webpage. Perhaps use the replacement?

We thank the reviewer for pointing this out, we have updated PyFPDF to FPDF2, which is the current version.
